# Early Detection and High‐Risk Monitoring of Cell‐Based Genetic Subtypes Using Plasma Biomarkers

**DOI:** 10.1002/alz70856_103575

**Published:** 2025-12-25

**Authors:** Nathan J Sahelijo, Daniel Goldstein, Gyungah R Jun

**Affiliations:** ^1^ Boston University School of Medicine, Boston, MA, USA; ^2^ Boston University Chobanian & Avedisian School of Medicine, Boston, MA, USA; ^3^ Biomedical Genetics Section, Department of Medicine, Boston University Chobanian & Avedisian School of Medicine, Boston, MA, USA

## Abstract

**Background:**

Genetic subtyping can identify subpopulations at risk for developing Alzheimer Disease (AD), but it cannot detect definitive disease progression. Plasma biomarkers enable a cost‐effective, noninvasive approach to early detection of AD disease.

**Method:**

We defined genetic subtypes by calculating cell‐based polygenic risk scores (cbPRSs) with a suggestive significance (*p* < 1×10^‐5^) in 8,481subjects from the Framingham Heart Study (FHS) using previously described procedures (Sahelijo et al., 2023). We defined high‐ and low‐risk individuals for four cell‐specific subtypes using the first and fourth quartiles of the cbPRS distribution in FHS. We analyzed differential expression of 1377 SOMAscan aptamer‐based plasma proteins between high‐ and low‐risk individuals in FHS using the LIMMA R software package, adjusting for age and sex. Pathway analysis was conducted for nominally significant differentially expressed proteins (DEP) with *p* <0.05 using Metascape software. Finally, we validated the identified subtype‐specific DEPs using clinically or neuropathologically defined AD diagnosis in autopsied brains from Boston University Alzheimer's Disease Research Center to identify the overlap of the subtype DEP with AD DEP using gene set enrichment analysis (GSEA).

**Result:**

Genetic subtypes were defined for two astrocyte and two oligodendrocyte networks with low and high‐risk sample sizes ranging from 2,072 to 2,090 subjects in FHS. Differential expression analysis revealed 59 to 91 nominally significant proteins with some overlapping proteins among the subtypes. All four subtypes exhibited enrichment for pathways associated with cytokine regulation and immune response. Top plasma DEP for Ast‐M2 was the MMP‐8 protein, which was validated in brain DEP for clinical and pathological AD (p_AstM2_= 4.63×10^‐5^, p_clinicalAD_=9.98×10^‐5^, p_PathAD_=3.2×10^‐3^). Moreover, using GSEA we identified significant enrichment of DEP in clinical AD with Ast‐M2 DEP (p_AstM2_=1.30×10^‐2^) and pathological AD with Ast‐M10 DEP (p_AstM10_=3.72×10^‐4^).

**Conclusion:**

Plasma protein levels from at‐risk individuals for cell‐based genetic subtypes exhibited considerable enrichment for neuroinflammatory proteins, indicating potential as genetic‐subtype‐specific biomarkers. These results highlight plasma biomarkers as cost‐effective tools for early detection in genetically at‐risk individuals of specific brain cell types.

